# Genetic associations between Transcription Factor 7 Like 2 rs7903146 polymorphism and type 2 diabetes mellitus: a meta-analysis of 115,809 subjects

**DOI:** 10.1186/s13098-019-0451-9

**Published:** 2019-07-05

**Authors:** Liying Lou, Jingjing Wang, Jing Wang

**Affiliations:** Department of Endocrinology, Shengzhou People’s Hospital, No. 666 Dangui Road of Sanjiang Street, Shaoxing, 312400 Zhejiang China

**Keywords:** Transcription Factor 7 Like 2 (*TCF7L2*), rs7903146 polymorphism, Type 2 diabetes mellitus (T2DM), Meta-analysis

## Abstract

**Background:**

Some genetic association studies tried to investigate potential associations of Transcription Factor 7 Like 2 (*TCF7L2*) rs7903146 polymorphism with type 2 diabetes mellitus (T2DM). However, the results of these studies were not consistent. Thus, we performed the present meta-analysis to explore associations between *TCF7L2* rs7903146 polymorphism and T2DM in a larger pooled population.

**Methods:**

Systematic literature research of PubMed, Web of Science and Embase was performed to identify eligible studies for pooled analyses. I^2^ statistics were employed to assess between-study heterogeneities. If I^2^ was greater than 50%, random-effect models (REMs) would be used to pool the data. Otherwise, fixed-effect models (FEMs) would be applied for synthetic analyses.

**Results:**

Totally 68 studies with 115,809 subjects were included for analyses. The pooled analyses showed that *TCF7L2* rs7903146 (dominant model: *p* < 0.0001; recessive model: *p* < 0.0001; over-dominant model: *p* < 0.0001; allele model: *p* < 0.0001) polymorphism was significantly associated with susceptibility to T2DM in overall population. Further subgroup analyses revealed similar significant findings in both Asians and Caucasians.

**Conclusions:**

In conclusion, our findings supported that *TCF7L2* rs7903146 polymorphism could be used to identify individuals at high risk of developing T2DM in Asians and Caucasians.

**Electronic supplementary material:**

The online version of this article (10.1186/s13098-019-0451-9) contains supplementary material, which is available to authorized users.

## Background

Type 2 diabetes mellitus (T2DM), characterized by chronic hyperglycemia caused by insufficient responses to insulin, is the most prevalent type of metabolic disorder, and it is estimated that over 344 million people are currently affected by this disease worldwide [1, 2]. So far, the exact pathogenesis of T2DM is still not fully understood. However, past genome-wide association studies already identified over 100 genetic loci that were significantly associated with an increased susceptibility to T2DM, which supported that inherit factors were crucial for its occurrence and development [3, 4].

Transcription Factor 7 Like 2 (*TCF7L2*) gene encodes T cell transcription factor 4, a transcription factor of the Wnt/β-catenin signaling pathway that is vital for embryogenesis of the pancreas islet and regulation of blood glucose [5, 6]. Recently, some genome-wide association studies found that *TCF7L2* rs7903146 polymorphism could significantly affect individual susceptibility to T2DM in certain populations [7, 8]. Since then, many genetic association studies were performed in diverse populations to estimate potential associations between *TCF7L2* rs7903146 polymorphism and T2DM, with inconsistent results. In 2018, Ding et al. [9] already performed a meta-analysis to assess association between *TCF7L2* rs7903146 polymorphism and T2DM, but only 28 studies were included by the authors and many eligible studies were missed. Therefore, we conducted an updated meta-analysis of all relevant studies published before May 2019 to more comprehensively analyze the effects of *TCF7L2* rs7903146 polymorphism on individual susceptibility to T2DM in a larger pooled population.

## Methods

The current meta-analysis was reported according to the Preferred Reporting Items for Systematic Reviews and Meta-analyses (PRISMA) statement [10].

### Literature search and inclusion criteria

Potentially relevant articles were searched in PubMed, Medline and Web of Science using the following key words: “TCF7L2”, “Transcription Factor 7 Like 2”, “polymorphism”, “variant”, “mutation”, “SNP”, “genotype”, “allele”, “type 2 diabetes”, “type II diabetes” and “T2DM”. The initial literature search was performed in January 2019 and the latest update was finished in May 2019. Moreover, we also screened the references of all retrieved articles to identify other potential relevant studies.

Included studies must meet all the following criteria: (1) genetic association studies on associations between *TCF7L2* rs7903146 polymorphism and T2DM in human beings; (2) provide genotypic/allelic frequency of *TCF7L2* rs7903146 polymorphism in cases and controls; (3) full text in English available. For duplicate reports, only the most complete one was included. Studies were excluded if one of the following criteria was fulfilled: (1) not about *TCF7L2* rs7903146 polymorphism and T2DM; (2) studies that were not performed in human beings; (3) case reports or case series; (4) reviews, comments and conference presentations.

### Data extraction and quality assessment

The following data were extracted from included studies: (1) Last name of first author; (2) Year of publication; (3) Country where the study was conducted and ethnicity of study participants; (4) type of disease; (5) the number of cases and controls; and (6) genotypic/allelic distributions of *TCF7L2* rs7903146 polymorphism in cases and controls. The probability value (*p* value) of Hardy–Weinberg equilibrium (HWE) was also calculated. When necessary, we wrote to the corresponding authors for extra information. We used the Newcastle–Ottawa scale (NOS) to assess the quality of eligible studies [11]. This scale has a score range of zero to nine, and studies with a score of more than seven were thought to be of high quality. Data extraction and quality assessment were performed by two independent reviewers. Any disagreement between two reviewers was solved by discussion until a consensus was reached.

### Statistical analyses

We used Review Manager Version 5.3.3 (The Cochrane Collaboration, Software Update) to conduct statistical analyses. We calculated odds ratios (ORs) and 95% confidence intervals (CIs) to estimate strength of associations between *TCF7L2* rs7903146 polymorphism and T2DM in dominant, recessive, over-dominant and allele models. Statistical significances of pooled analyses were determined by the Z test, with a *p* value of 0.05 or less was defined as statistically significant. I^2^ statistics were employed to assess between-study heterogeneities. If I^2^ was greater than 50%, random-effect models (REMs) would be used to pool the data on account of significant heterogeneities. Otherwise, fixed-effect models (FEMs) would be used for synthetic analyses. Subgroup analyses by ethnicity of participants were subsequently performed to evaluate effects of ethnic background on investigated genetic associations. Sensitivity analyses were carried out to test the stability of pooled results by omitting one study each time and re-perform analyses based on the results of the remaining studies. Publication biases were evaluated with funnel plots.

## Results

### Characteristics of included studies

**Fig. 1:**
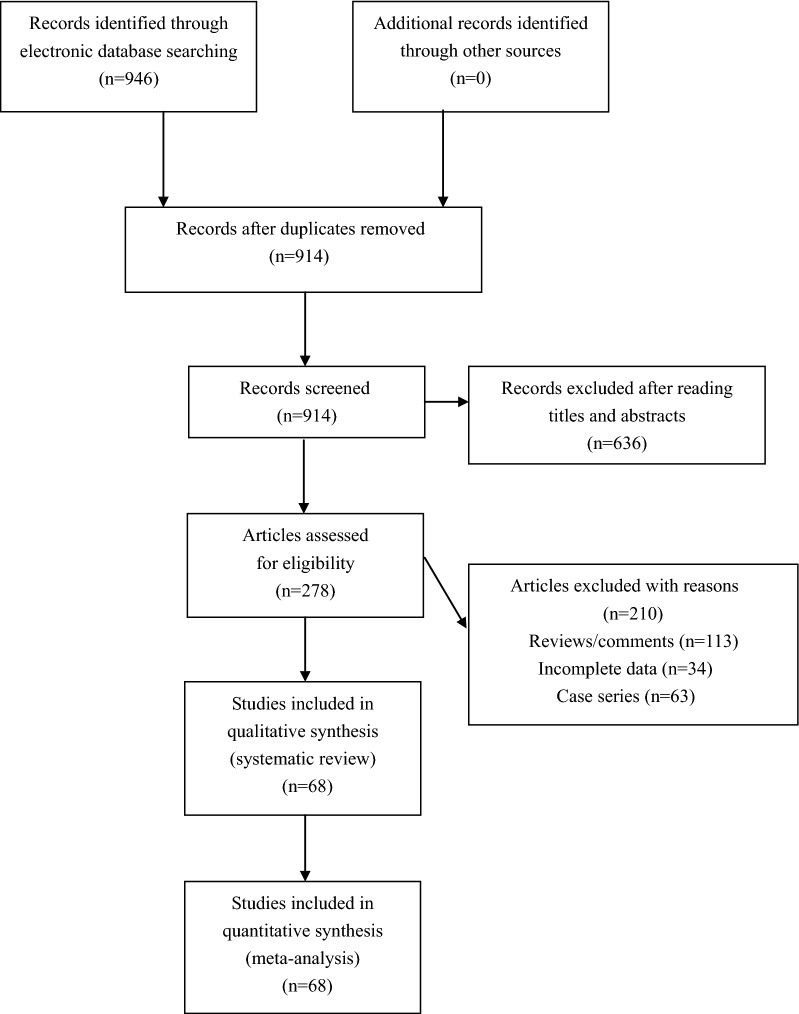
Flowchart of study selection for the present study

The initial literature search found 946 potential relevant articles. After exclusion of irrelevant and duplicate articles by reading titles and abstracts, 278 potentially relevant articles were retrieved for eligibility assessment. Another 210 articles were subsequently excluded after reading the full text. Finally, a total of 68 studies that met the inclusion criteria of our meta-analysis were included (Fig. 1). Baseline characteristics of included studies were shown in Table 1.

**Table 1:** The characteristics of included studies

First author, year	Country	Ethnicity	Type of disease	Sample size	Genotypes (wtwt/wtmt/mtmt)		*p* value for HWE	NOS score
					Cases	Controls		
rs7903146 C/T								
Acharya 2015	Saudi Arabia	South Asian	T2DM	359/351	131/137/91	132/143/76	0.002	8
Al-Sinani 2015	Oman	South Asian	T2DM	992/294	NA	NA	NA	7
Anjum 2018	China	East Asian	T2DM	339/191	160/117/62	110/56/25	< 0.001	7
Assmann 2014	Brazil	Mixed	T2DM	953/535	382/415/156	261/215/59	0.147	8
Barra 2012	Brazil	Mixed	T2DM	113/139	49/47/17	70/63/6	0.076	7
Barros 2014	Brazil	Mixed	T2DM	108/109	53/49/6	58/40/11	0.304	7
Beloso 2018	Uruguay	Mixed	T2DM	177/133	84/66/27	71/47/15	0.104	7
Bielicki 2019	Poland	Caucasian	T2DM	121/479	69/45/7	285/172/22	0.539	7
Bodhini 2007	India	South Asian	T2DM	1031/1038	462/455/114	555/391/92	0.055	8
Cai 2019	China	East Asian	T2DM	296/446	197/83/16	287/147/12	0.180	8
Cauchi 2006	France	Caucasian	T2DM	2367/2499	787/1149/431	1208/1060/231	0.944	8
Chandak 2007	India	South Asian	T2DM	955/399	391/423/141	205/160/34	0.726	8
Chang 2007	Taiwan	East Asian	T2DM	760/760	NA	NA	NA	7
Chidambaram 2016	India	South Asian	T2DM	877/838	NA	NA	NA	7
Corella 2016	Spain	Caucasian	T2DM	3411/3607	1158/1680/573	1612/1569/426	0.140	8
Dahlgren 2017	Sweden	Caucasian	T2DM	168/885	67/83/18	496/327/62	0.421	8
Danquah 2013	Germany	Caucasian	T2DM	674/375	273/323/78	182/165/28	0.257	7
De Silva 2007	UK	Caucasian	T2DM	601/2099	211/299/91	1032/887/180	0.586	7
El-Lebedy 2016	Egypt	Caucasian	T2DM	180/210	48/126/6	112/95/3	< 0.001	8
Erkoç Kaya 2017	Turkey	Caucasian	T2DM	171/120	58/95/18	57/47/16	0.215	7
Ezzidi 2009	Tunisia	Caucasian	T2DM	863/511	250/396/217	181/235/95	0.227	8
Groves 2006	UK	Caucasian	T2DM	2001/2476	771/960/270	1175/1084/217	0.139	8
Guewo-Fokeng 2015	Cameroon	African	T2DM	74/74	37/30/7	37/37/0	0.004	7
Gupta 2010	India	South Asian	T2DM	195/161	55/96/44	62/78/21	0.647	8
Hayashi 2007	Japan	East Asian	T2DM	1619/1069	1450/165/4	980/85/2	0.146	8
Horikoshi 2007	Japan	East Asian	T2DM	1174/823	1051/119/4	770/51/2	0.243	8
Hsiao 2017	Taiwan	East Asian	T2DM	562/986	497/62/3	933/52/1	0.755	7
Humphries 2016	UK	Caucasian	T2DM	1459/2493	601/665/193	1295/1001/197	0.854	7
Humphries 2016	UK	South Asian	T2DM	837/300	366/375/96	163/111/26	0.260	7
Humphries 2016	UK	African	T2DM	307/311	141/136/30	161/124/26	0.759	7
Hussain 2014	India	South Asian	T2DM	123/82	45/63/15	43/35/4	0.350	7
Isakova 2019	Kyrgyzstan	Caucasian	T2DM	114/109	91/20/3	89/16/4	0.009	8
Jia 2016	China	East Asian	T2DM	248/267	125/73/50	165/74/28	< 0.001	8
Kalantari 2019	Iran	South Asian	T2DM	530/420	155/241/134	187/173/60	0.056	7
Katsoulis 2018	Greece	Caucasian	T2DM	148/80	30/104/14	54/23/3	0.779	7
Khan 2015	India	South Asian	T2DM	42/98	13/18/11	57/33/8	0.312	7
Khan 2015	India	South Asian	T2DM	250/250	92/120/38	144/87/19	0.255	7
Kimber 2007	UK	Caucasian	T2DM	3225/3291	1405/1459/361	1714/1329/248	0.663	8
Kong 2015	China	East Asian	T2DM	5169/4560	NA	NA	NA	7
Kunika 2008	Japan	East Asian	T2DM	1422/1423	1246/171/5	1309/111/3	0.689	8
Löfvenborg 2019	Sweden	Caucasian	T2DM	1242/1530	NA	NA	NA	7
Marquezine 2008	Brazil	Mixed	T2DM	285/1681	83/160/42	684/833/164	< 0.001	8
Mayans 2007	Sweden	Caucasian	T2DM	824/820	452/318/54	532/253/35	0.481	8
Miranda-Lora 2017	Mexico	Mixed	T2DM	156/212	115/38/3	157/51/4	0.952	8
Miyake 2008	Japan	East Asian	T2DM	2154/1834	1921/228/5	1696/137/1	0.295	8
Moran 2015	Venezuela	African	T2DM	70/73	26/35/9	46/22/5	0.307	8
Musavi 2015	Iran	South Asian	T2DM	70/100	19/36/15	45/48/7	0.222	7
Ouhaibi-Djellouli 2014	Algeria	African	T2DM	76/644	16/41/19	228/287/129	0.027	8
Palizban 2017	Iran	South Asian	T2DM	204/80	60/95/49	32/41/7	0.224	8
Palmer 2011	USA	Mixed	T2DM	982/1039	NA	NA	NA	7
Papandreou 2019	Spain	Caucasian	T2DM	869/244	382/383/104	106/103/35	0.225	8
Plengvidhya 2018	Thailand	East Asian	T2DM	500/500	429/67/4	456/44/0	0.303	8
Pourahmadi 2015	Iran	South Asian	T2DM	200/200	109/68/23	126/59/15	0.037	8
Rees 2008	UK	South Asian	T2DM	828/432	352/360/116	222/166/44	0.122	8
Reyes-López 2019	Mexico	Mixed	T2DM	23/83	14/6/3	59/24/0	0.124	7
Saadi 2008	United Arab Emirates	South Asian	T2DM	180/188	56/103/21	71/94/23	0.339	7
Scott 2006	USA	Mixed	T2DM	1151/953	NA	NA	NA	7
Tabara 2009	Japan	East Asian	T2DM	481/398	434/45/2	372/26/0	0.501	8
Turki 2013	Tunisia	South Asian	T2DM	895/878	255/432/208	330/414/134	0.824	7
Uma Jyothi 2015	India	South Asian	T2DM	758/621	341/326/83	391/193/37	0.048	7
van Vliet-Ostaptchouk 2007	Netherlands	Caucasian	T2DM	496/907	203/221/72	459/365/83	0.397	7
Včelák 2012	Czech Republic	Caucasian	T2DM	347/376	148/156/43	205/147/24	0.731	8
Wang 2013	China	East Asian	T2DM	1842/7777	1553/283/6	6718/1032/27	0.057	8
Wrzosek 2019	Poland	Caucasian	T2DM	129/345	67/50/12	219/113/13	0.738	8
Yako 2015	South Africa	African	T2DM	152/328	66/74/12	184/129/15	0.199	8
Yu 2009	USA	Mixed	T2DM	686/305	355/271/60	170/111/24	0.330	8
Zhang 2016	China	East Asian	T2DM	227/5284	200/24/3	4567/701/16	0.045	8
Zheng 2012	China	East Asian	T2DM	227/152	202/24/1	139/13/0	0.582	8
Zhu 2017	China	East Asian	T2DM	497/782	478/19/0	740/41/1	0.584	8
Zhuang 2018	China	East Asian	T2DM	90/96	54/26/10	69/24/3	0.611	7

*T2DM* type 2 diabetes mellitus, *wt* Wild type, *mt* mutant type, *HWE* Hardy–Weinberg equilibrium, *NOS* Newcastle–ottawa scale, *NA* not available

### TCF7L2 rs7903146 polymorphism and T2DM

The results of overall and subgroup analyses were summarized in Table 2. Totally 68 studies with 115,809 subjects were included for analyses, the pooled analyses showed that *TCF7L2* rs7903146 (dominant model: *p* < 0.0001, OR = 0.66, 95% CI 0.63–0.70; recessive model: *p* < 0.0001, OR = 1.64, 95% CI 1.56–1.73; over-dominant model: *p* < 0.0001, OR = 1.27, 95% CI 1.21–1.34; allele model: *p* < 0.0001, OR = 0.71, 95% CI 0.68–0.74) polymorphism was significantly associated with susceptibility to T2DM in overall population. Further subgroup analyses revealed similar significant findings in both Asians and Caucasians (Table 2).

**Table 2:** Results of overall and subgroup analyses

Variables	Sample size	Dominant comparison	Recessive comparison	Over-dominant comparison	Allele comparison
		*p* value OR (95% CI)	*p* value OR (95% CI)	*p* value OR (95% CI)	*p* value OR (95% CI)
Overall	51,656/64,153	*<* *0.0001 0.66 (0.63–0.70)*	*<* *0.0001 1.64 (1.56–1.73)*	*<* *0.0001 1.27 (1.21–1.34)*	*<* *0.0001 0.71 (0.68–0.74)*
Caucasian	19,410/23,456	*<* *0.0001 0.64 (0.58–0.70)*	*<* *0.0001 1.64 (1.54–1.75)*	*<* *0.0001 1.31 (1.21–1.43)*	*<* *0.0001 0.70 (0.65–0.75)*
East Asian	17,607/27,348	*<* *0.0001 0.73 (0.63–0.83)*	*<* *0.0001 1.90 (1.46–2.46)*	*0.0006 1.28 (1.11–1.48)*	*<* *0.0001 0.74 (0.66–0.83)*
South Asian	9326/6730	*<* *0.0001 0.63 (0.59–0.68)*	*<* *0.0001 1.65 (1.48–1.84)*	*<* *0.0001 1.24 (1.16–1.33)*	*<* *0.0001 0.65 (0.60–0.71)*

*OR* odds ratio, *CI* confidence interval, *NA* not available, *T2DM* type 2 diabetes mellitus

### Sensitivity analyses

We performed sensitivity analyses by deleting one individual study each time to test the effects of individual study on pooled results. No any altered results were observed in overall and subgroup comparisons, which indicated that our findings were statistically robust.

### Publication biases

We used funnel plots to assess publication biases. We did not find obvious asymmetry of funnel plots in any comparisons, which suggested that our findings were unlikely to be impacted by severe publication biases (Additional file 1: Fig. S1).

## Discussion

Despite prominent advancements achieved in drug therapy over the last few decades, T2DM and its associated vascular complications are still leading causes of death and disability around the world [12, 13]. The exact cause of T2DM is still largely unclear in spite of extensive explorations. However, the obvious familial aggregation tendency of T2DM indicated that genetic factors played significant parts in its pathogenesis [14]. Thus, identify genetic biomarkers is of particularly importance for an early diagnosis and a better prognosis of T2DM patients.

TCF7L2, a box-containing transcription factor that is vital for blood glucose homeostasis, is considered to act through regulation of proglucagon gene expression in enteroendocrine cells via the Wnt signaling pathway [15], and pre-clinical studies also found that TCF7L2 expression is positively associated with insulin gene expression in human islets [16]. Considering the vital role of TCF7L2 in regulating blood glucose, many genetic association studies were performed in diverse populations to investigate whether functional *TCF7L2* polymorphisms could impact individual susceptibility to T2DM. To our knowledge, this is to date the most comprehensive meta-analysis on association between *TCF7L2* rs7903146 polymorphism and T2DM, and our pooled analyses suggested that *TCF7L2* rs7903146 polymorphism was significantly associated with T2DM in both Asians and Caucasians. The stabilities of synthetic results were evaluated by sensitivity analyses, and no alterations of results were observed in any comparisons, which suggested that our findings were statistically robust. Significant heterogeneities were detected for dominant and allele comparisons, thus pooled analyses for these two genetic models were performed with REMs. But in further subgroup analyses, an obvious reduction tendency of heterogeneity was found in both Asians and Caucasians, which suggested that differences in ethnic background could largely explain observed heterogeneities between studies. Nevertheless, it is worth noting that the obvious heterogeneities existed among included studies indicated that the distribution of *TCF7L2* rs7903146 polymorphism varies greatly from population to population. Therefore, the genetic association between *TCF7L2* rs7903146 polymorphism and T2DM may be ethnic-specific, and we should not generalize the subgroup analyses results to a broader population.

There are several points that need to be pointed out about the current study. First, the exact underlying molecular mechanisms of our positive findings remains to be explored, but we speculated that *TCF7L2* rs7903146 polymorphism may lead to alternations in gene expression or changes in protein structure, which may subsequently affect biological functions of TCF7L2, impact insulin secretion or decrease sensitivity to insulin, and ultimately affect individual susceptibility to T2DM. Second, the pathogenic mechanism of T2DM is extremely complex, and hence despite our positive findings, it is unlikely that a single gene polymorphism could significantly contribute to its development, and thus we strongly recommend further studies to perform haplotype analyses and explore potential gene–gene interactions [17, 18]. Third, to more precisely measure the effects of certain genetic factors on disease occurrence and development, gene-environmental interactions should also be considered. However, since included studies only focused on the effects of *TCF7L2* rs7903146 polymorphism on individual susceptibility to T2DM, such analyses were not applicable in the current meta-analysis. But to better elucidate the underlying pathogenesis mechanisms of T2DM, future studies should try to investigate the interaction of *TCF7L2* gene polymorphisms with potential pathogenic environmental factors such as unhealthy diets or lack of exercise [19]. Our meta-analysis certainly has some limitations. Firstly, although methodology qualities of included studies were generally good, it should be noted that we did not have access to genotypic distributions of investigated polymorphisms according to base characteristics of study subjects. Therefore, our results were derived from unadjusted estimations, and failure to conduct further adjusted analyses for baseline characteristics of participants such as age, gender and co-morbidity conditions may influence the veracity of our findings [20, 21]. Secondly, significant heterogeneities were detected in certain subgroup comparisons, which indicated that the inconsistent results of included studies could not be fully explained by differences in ethnic background, and other unmeasured characteristics of participants may also partially attribute to between-study heterogeneities [22]. Thirdly, since only published articles were eligible for analyses, although funnel plots revealed no obvious publication biases, we still could not rule out the possibility of potential publication biases [23]. Taken these limitations into consideration, the results of the current study should be interpreted with caution.

## Conclusions

In conclusion, our findings indicated that *TCF7L2* rs7903146 polymorphism was significantly associated with altered susceptibility to T2DM in both Asians and Caucasians. These results supported that this polymorphism may be used to identify individuals at high risk of developing T2DM in Asians and Caucasians. Further well-designed studies need to explore possible associations between other *TCF7L2* gene polymorphisms and T2DM.

## Additional file

**Additional file 1: Figure S1.** Funnel plots.

## Data Availability

The current study was based on results of relevant published studies.

## Abbreviations

*TCF7L2*: Transcription Factor 7 Like 2
T2DM: type 2 diabetes mellitus
HWE: Hardy–Weinberg equilibrium
NOS: Newcastle–Ottawa scale
REM: random-effect model
FEM: fixed-effect model
