# Supplementary material for: Genetic associations between Transcription Factor 7 Like 2 rs7903146 polymorphism and type 2 diabetes mellitus: a meta-analysis of 115,809 subjects
Source: Diabetol Metab Syndr. 2019 Jul 5;11:56. doi: 10.1186/s13098-019-0451-9 (PMC6612193; doi:10.1186/s13098-019-0451-9)
Supplement: Supplementary file 1 — Additional file 1: Figure S1. Funnel plots. [file 13098_2019_451_MOESM1_ESM.docx]

**Additional file 1: figure S1. Funnel plots**


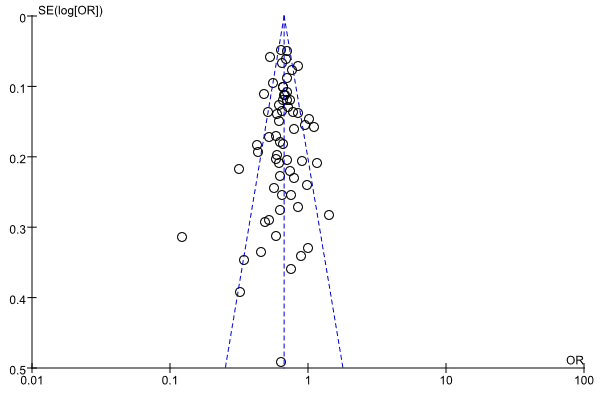


Funnel plot of the rs7903146 polymorphism and T2DM under dominant comparison.


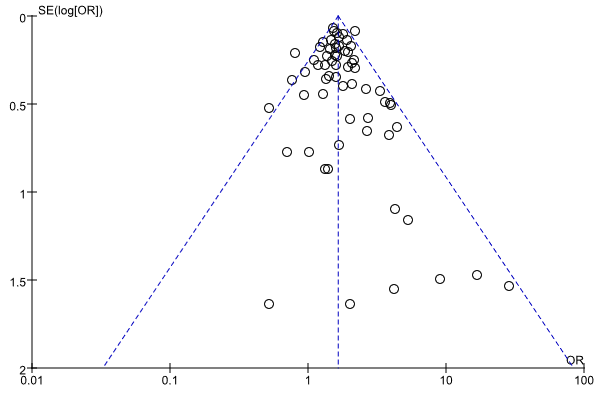


Funnel plot of the rs7903146 polymorphism and T2DM under recessive comparison.


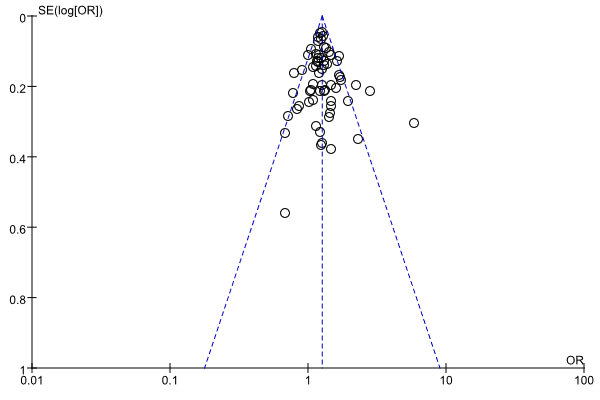


Funnel plot of the rs7903146 polymorphism and T2DM under over-dominant comparison.


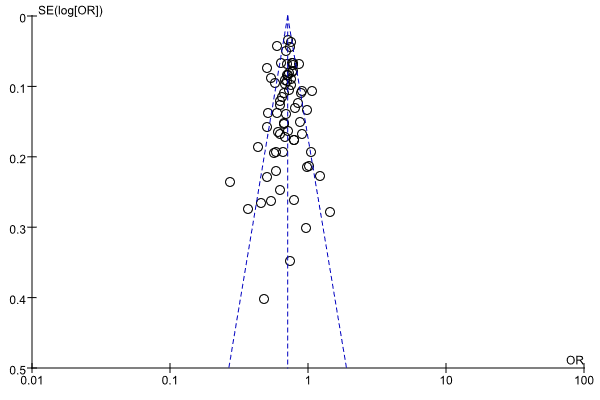


Funnel plot of the rs7903146 polymorphism and T2DM under allele comparison.
